# Physiotherapy in Patients with Stress Urinary Incontinence: A Systematic Review and Meta-analysis

**DOI:** 10.5152/tud.2023.23018

**Published:** 2023-09-01

**Authors:** Fariba Ghaderi, Ghazal Kharaji, Sakineh Hajebrahimi, Fariba Pashazadeh, Bary Berghmans, Hanieh Salehi Pourmehr

**Affiliations:** 1 Department of Physiotherapy, Faculty of Rehabilitation Sciences, Tabriz University of Medical Sciences, Tabriz, Iran; 2 Department of Physiotherapy, Faculty of Rehabilitation Sciences, Iran University of Medical Sciences, Tehran, Iran; 3 Research Center for Evidence-Based Medicine, Iranian EBM Centre: A JBI Centre of Excellence, Tabriz University of Medical Sciences, Faculty of Medicine, Tabriz, Iran; 4 Pelvic Care Unit Maastricht, CAPHRI, Maastricht University Medical Centre (MUMC+), Maastricht, The Netherlands; 5 Research Center for Evidence-Based Medicine, Iranian EBM Center: A Joanna Briggs Institute Center of Excellence, Tabriz University of Medical Sciences, Tabriz, Iran

**Keywords:** Pelvic floor, physiotherapy, urinary incontinence, stress, urgency, mixed, systematic review

## Abstract

Physiotherapy is the most commonly used treatment for stress urinary incontinence including pelvic floor muscle training, biofeedback, and electrical stimulation. This systematic review evaluated the effects of physiotherapy in patients with stress urinary incontinence compared with no treatment, placebo, sham, surgery, or other inactive control treatments. MEDLINE (via PubMed), The Cochrane Library (CENTRAL), Embase, Scopus, Web of Science, PEDro, and Trip Database were explored using applicable vocabularies for all English and Persian language investigations released from inception to January 2021. On one side, trials including physiotherapy of pelvic floor muscle training, biofeedback, and electrical stimulation and on the other, either no treatment, placebo, sham, surgery, or other inactive control treatments were included. Studies were assessed for appropriateness and methodological excellence. Two authors extracted data. Disagreements were resolved by a third opinion. Data were processed as described in the *Joanna Briggs Institute* *Handbook*. Twenty-nine trials with 2601 participants were found, but only 16 were included because of data heterogeneity. The results showed that physiotherapy interventions are better than no treatment in terms of urine leakage, but no difference was found for urinary incontinence severity. Also, physiotherapy showed favorable results over comparison groups for International Consultation on Incontinence Questionnaire, pad test, pelvic floor muscle function, and improvement outcomes. This systematic review supports the widespread use of pelvic physiotherapy as the first-line treatment for adult patients with stress urinary incontinence.

**Main Points**

- The pelvic floor muscles and urogenital diaphragm have the essential role to keep the urethra closed when pressure is placed on the bladder.
- Pelvic floor muscles training is the most important part of physiotherapy for preventing and treating stress urinary incontinence
- More and valid randomized controlled trial studies are needed to have better systematic reviews and draw conclusions about other physiotherapy methods.

## Introduction

According to the Sixth International Consultation on Incontinence (ICI), stress urinary incontinence (SUI) is the unconscious loss of urine during physical exertion, sneezing, and coughing that often results in impaired quality of life (QOL), personal hygiene, and social relationships.^1^ The prevalence of SUI is 24.8% in the United States^2^ and 57.7% in Iran.^3^

In 25% of patients, SUI has negative impacts on various aspects of life including social, psychological, occupational, physical,^4,5^ and sexual activities.^6^ Besides, UI has a substantial financial impact on people's lives.^7^

Several tests are used to diagnose and monitor urinary incontinence (UI), either subjective or objective.^8^ The International Consultation on Incontinence Questionnaire on Female Urinary Tract Symptoms (ICIQ-FLUTS), the Urogenital Distress Inventory 6 (UDI), the Incontinence Impact Questionnaire—Short Form (ICIQ-SF),^1,9^ and the King’s Health Questionnaire are the most frequently used tools to assess incontinence impact on daily life.^10^ As functional tests, physical assessment, dynamometer, electromyography, ultrasound and magnetic resonance imaging,^11^ a bladder diary,^12^ and pad tests^13^ are regularly used in UI evaluations.

At present, there are surgical and conservative interventions for SUI. Physiotherapy is one of the most prescribed conservative treatments. Pelvic floor muscle training (PFMT), biofeedback (BF) therapy, electrical stimulation (ES), and vaginal weights are some of physiotherapy options.^14^

Pelvic floor muscle training is known as one of the first-line options for SUI.^15^ Pelvic floor muscle exercises (PFME) are used to increase (maximal) strength, endurance, timing, explosive strength, and muscle coordination. Pelvic floor muscle training includes passive, active-assisted, active-resisted, and simple contraction exercises with or without ES, BF therapy, and vaginal weights.^9,16^ Also, PFMT can be prescribed home-based or supervised, which are different in terms of adherence and compliance.^17^ Moreover, PFMT is used in combination with stabilization exercises to reduce SUI and lower back pain (LBP) symptoms in pregnant^18^ or elderly women.^19^

Biofeedback therapy is an adjunct to PFMT, a technique assessing physiologic processes of the body, which can be used to learn control some of body’s functions, such as activity of the pelvic floor muscles (PFMs). In the case of SUI, depending on the type of BF (EMG, pressure, or ultrasound), BF therapy makes patients aware of the activity of their PFMs through electromyographic activity, manometric squeeze pressure, or bladder base displacement.^20^

The scientific evidence for BF therapy in SUI treatment is still inconclusive probably due to different treatment methods (frequency of treatment sessions, type of contraction, and duration of contraction) in various studies.^21^ Electrical stimulation is another treatment option for SUI that includes suprapubic, transvaginal, sacral, and tibial nerve stimulation.^22,23^

Vaginal weights are used to train PFMs by inserting a weight into the vagina and asking the patient to hold it there by contracting the PFM. Once the patient succeeds to hold a certain weight, the next step is to replace the weights with a similar-sized but heavier one.^20^

To the best of our knowledge, few systematic reviews with meta-analyses have investigated the effectiveness of PFMT, alone or in combination with BF, ES, vaginal weights, or other types of exercises.^9,21,24^ The lack of consensus on the effects of these treatments and absence of an updated systematic review since 2018^24^ necessitated this systematic review. Therefore, this systematic review evaluated the effects of physiotherapy on SUI, episodes of urinary loss, quality of life (QOL), and muscle strength in adult women with SUI, compared with no treatment, placebo, sham, and surgery.

### Inclusion Criteria

#### Participants:

Studies that included adult women with SUI or mixed urinary incontinence with SUI as a dominant factor.

#### Intervention:

Physiotherapy involving PFME with or without BF, education and information, surface and intracavity ES, dynamic lumbopelvic stabilization exercises, magnetic stimulator, neuromuscular external stimulation device (NMES), and bladder training (BT).

#### Comparator:

No treatment, placebo, sham, surgery, or other inactive control treatments.

#### Outcomes:

Studies using measures such as pad test, Oxford Scale, PERFECT scale, manometry, EMG-BF, urodynamic investigation, UI leakage episodes, UI improvement based on validated measurements (ICIQ-FLUTS, UDI-6, ICIQ-SF, Patient Global Impression of Improvement (PGI-I)), and patient satisfaction were assessed to be included. Only randomized controlled trials were included. Studies published in English and Persian languages from inception to July 2021 were considered for inclusion.

## Material and Methods

This investigation was performed according to the Preferred Reporting Items for Systematic Reviews and Meta-Analyses (PRISMA)^25^ statement and the JBI methodology for systematic reviews. Besides, the study protocol was registered in* PROSPERO *registry for systematic reviews (**CRD42021233176**).

### Search Strategy

Published and unpublished evidence were searched. A three-step strategy was applied. First, a primary search was performed in PubMed and titles and abstracts were reviewed. Therefore, a comprehensive search was used using all identified keywords and index terms on January 2021 across MEDLINE (via PubMed), The Cochrane Library (CENTRAL), Embase, Scopus, Web of Science, PEDro, and Trip Database. Moreover, gray literature was searched in ProQuest (for theses), Google Scholar (for unpublished studies), and clinicaltrials.gov (for registered clinical trials). Finally, the reference lists of all selected documents were explored to find extra trials. The full search strategy for MEDLINE (via PubMed) and Embase is provided in Appendix I.

### Study Selection

All investigations were added into EndNote X7.1 (Clarivate Analytics). Duplicate studies were automatically removed. Titles and abstracts were investigated by 2 authors separately by considering the inclusion criteria. Thereafter, full texts of selected trials were read carefully. The reasons for excluding some investigations are illustrated in Appendix II. Any disagreements between the 2 authors were resolved by a third opinion.

### Assessment of Methodological Quality

Primarily included investigations were judiciously evaluated by two authors using tools from the Joanna Briggs Institute for experimental and quasi-experimental studies to determine methodological biases. Studies were categorized as low (11-13), moderate (8-10), and high risk (lower than 8) according to the consensus expert opinion. Studies with high risk of bias were excluded.

### Data Extraction

Data were extracted by two independent authors, using the modified standardized JBI data extraction tool (*Handbook of JBI for interventional Systematic Reviews*). Extracted data included authors and year of publication, intervention details including duration of treatment sessions and study, and sample size. Authors were contacted to request missing or additional data. Furthermore, in the case of unpublished trials, an e-mail was sent to the corresponding author(s) to ask whether the investigation was published. If no response received after 3 e-mails, the study was not included. 

### Data Synthesis

Where possible data were pooled using STATA v.14 (StataCorp, California, USA). Effect sizes, expressed as odds ratio (for categorical data), and weighted mean differences (for continuous data) and their 95% confidence intervals were calculated. Heterogeneity was evaluated by I^2^ tests. Statistical analyses were run using the random effect model.^26^ Subgroup analyses were performed where there was adequate data to examine based on manometry (cmH_2_O-mmHg) and pad test (long or short term). Publication bias was not assessed because there were less than 10 included studies. Despite that, tables and figures were designed when statistical pooling was not possible to help for further assessments.

### Results

#### Study Inclusion

**Figure 1.**
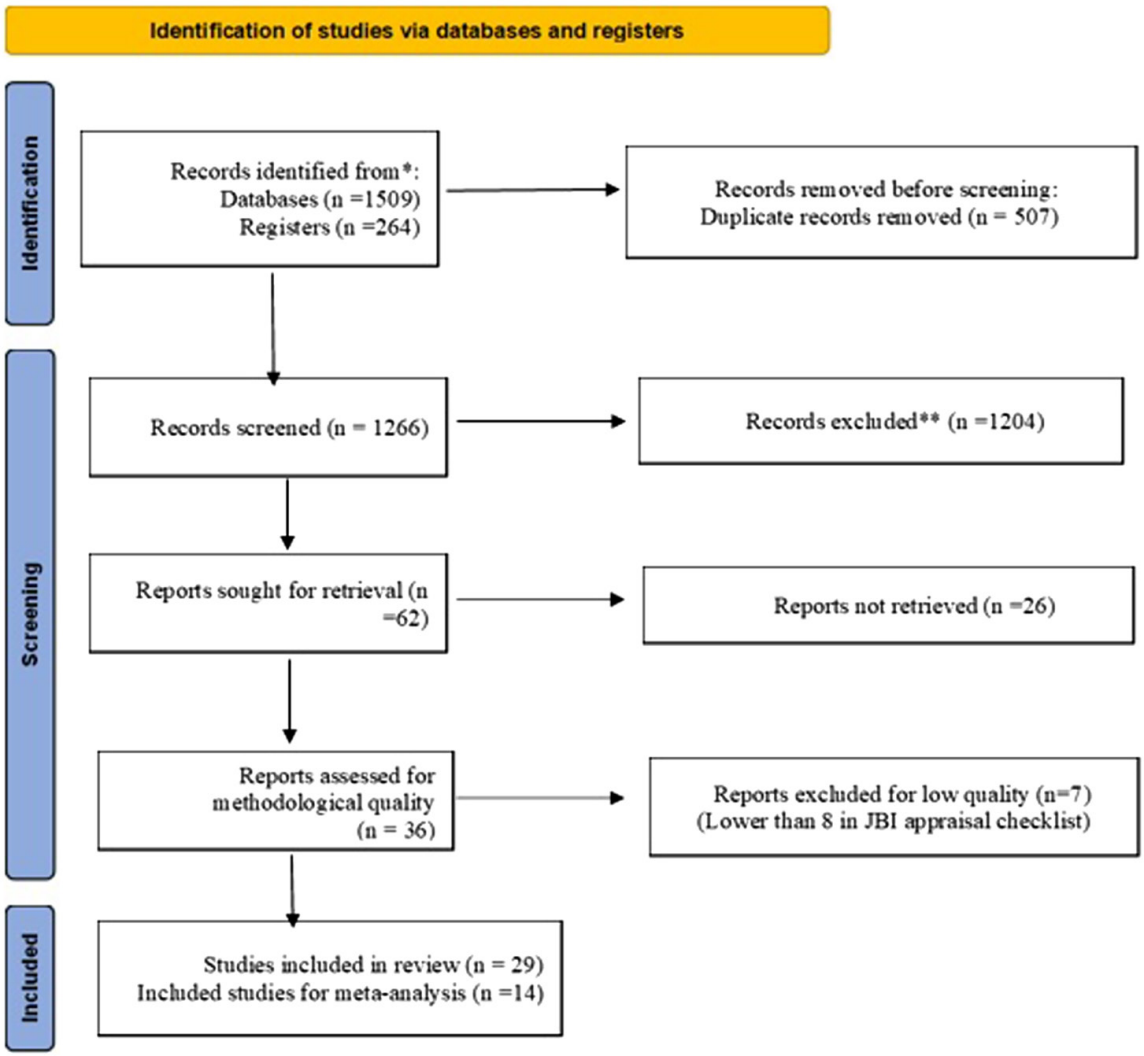
Search results, study selection, and inclusion process.

Totally, 1773 citations were identified by electronic search, hand search, and reference check. After removing duplicates, 1266 studies remained for the screening process. By reviewing titles and abstracts, 62 studies were selected. In the full-text selection, 23 studies were excluded. Finally, 36 studies were included for the critical appraisal process. Additional information on selection process is presented in the PRISMA flowchart (Figure 1).

### Summary of Included Articles

#### Methodological Quality:

Thirty-six eligible studies were critically appraised by the JBI appraisal checklists to assess possible biases. Twenty-nine studies were moderate (8-10 positive criteria) or high quality (11-13 positive criteria), and 7 were low quality (<8 positive criteria), which were excluded. Evaluation results of eligible studies are presented in Table 1.

### Characteristics of Included Studies

#### Participants:

Finally, 29 studies with 2601 patients were included in this systematic review and meta-analysis. Detailed information is presented in Table 2. All studies targeted women with SUI. Two studies included postnatal women,^17,27^ 2 postmenopausal women,^28,29^ 3 MUI,^30-32^ 1 volleyball athletes,^33^ and 1 overweight or obese elderly,^34^ and the others did not report any specific inclusion criteria. The sample size ranged from 14^35^ to 460.^35^

#### Interventions:

Physiotherapy treatments used in the selected studies presented different protocols. Studies performed PFMT with different protocols including PFMT alone or supplemented with education about urinary incontinence^31,33-46^ or with BF^28,47,48^ or ES,^19^ PFMT combined with stabilization exercises,^19,49^ transversus abdominis contraction,^50,51^ weight loss program,^30^ vaginal weights^29^ and occlusion training of a thigh,^52^ magnetic stimulators,^53^ intravaginal ES and BF without PFMT,^54-56^ and supporting underwear.^57^

#### Physiotherapy Versus No Treatment:

**Figure 2.**
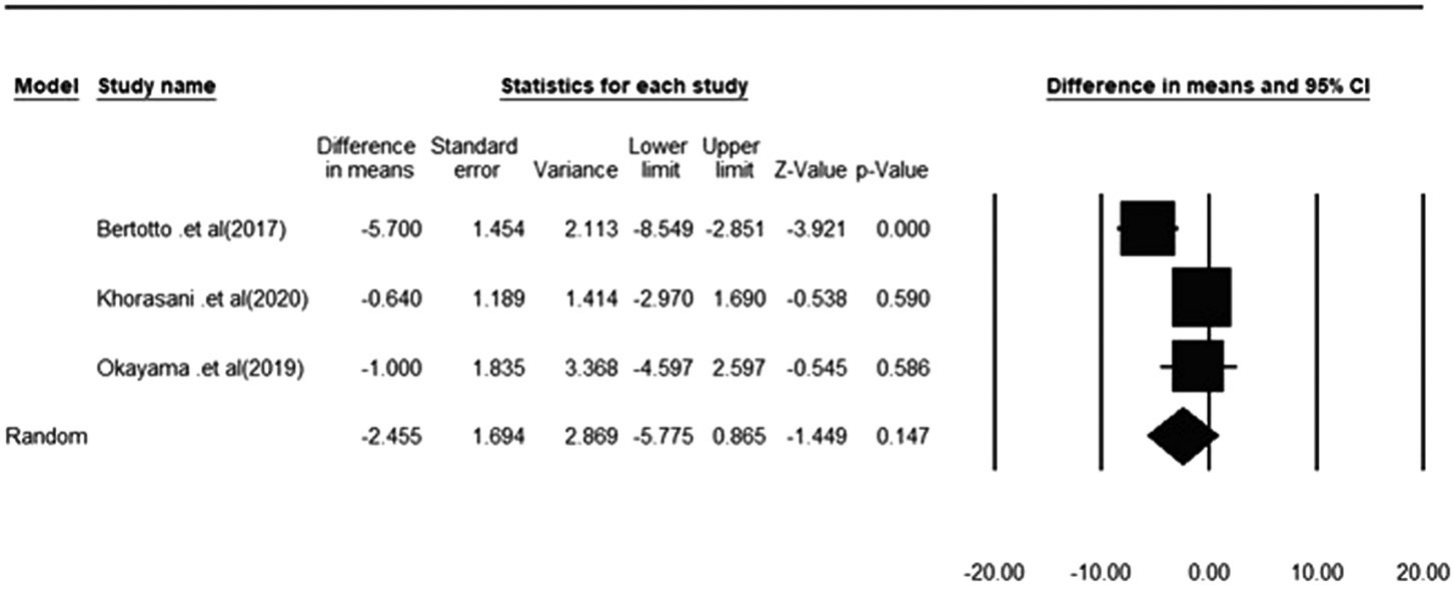
Difference between physiotherapy interventions and no treatment according to the ICIQ-SF. ICIQ-SF, International Consultation on Incontinence Questionnaire–Short Form.

**Figure 3.**
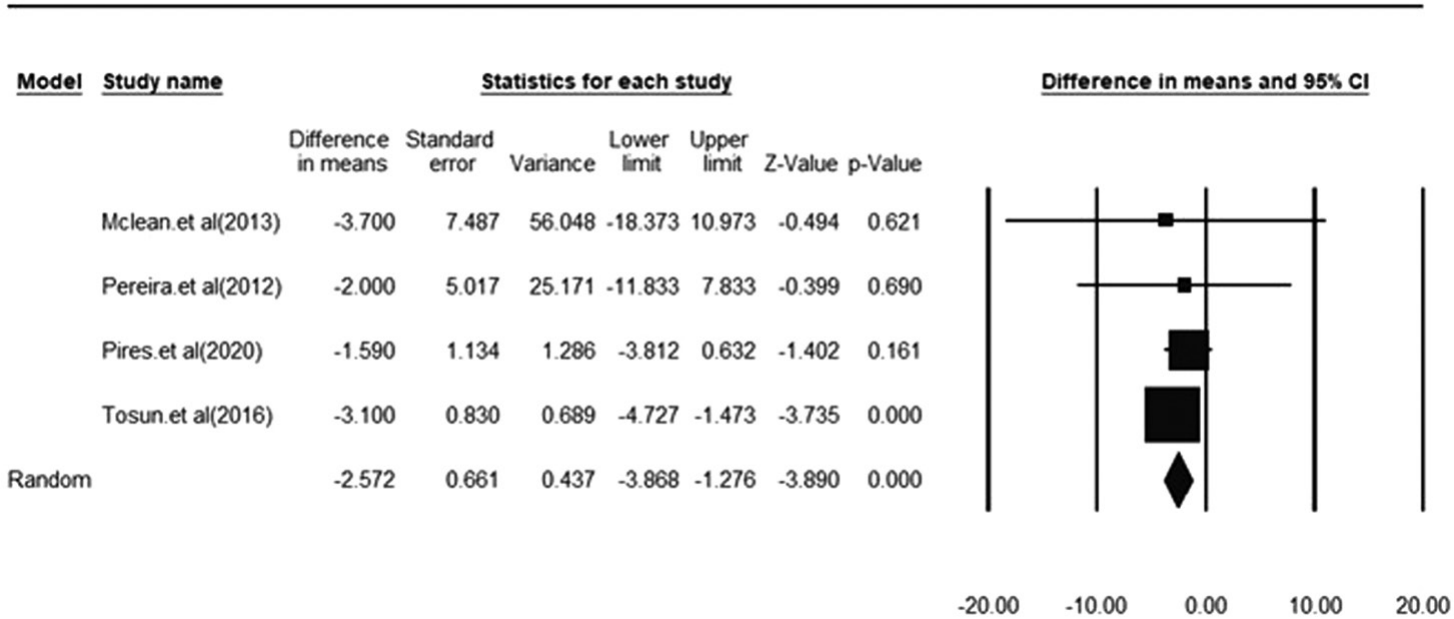
Difference between physiotherapy interventions and no treatment according to pad test.

Three studies compared physiotherapy interventions with no treatment in terms of UI severity using the ICIQ-SF. All of them showed favorable results for physiotherapy interventions over no treatment.^17,28,57^ All of these 3 studies were included in the meta-analysis. The results of the meta-analysis showed no differences between physiotherapy intervention and no-treatment groups (SMD: 1.69; 95% CI (−5.77, 0.87); *P* = 0.15) (Figure 2). In addition, 4 studies were found to compare physiotherapy interventions with no treatment using pad test, which were included in the meta-analysis.^29,33,37,41^ Physiotherapy interventions were found to be effective to improve urine leakage assessed by pad test, compared to no intervention (SMD: 0.66; 95% CI (−3.87, −1.28); *P* < .001) (Figure 3). Standard statistical tests for statistical heterogeneity indicated considerable statistical heterogeneity in UI severity assessed by the ICIQ-SF, among the included studies [I^2^: 74.75%; *P* = .02]. Statistical heterogeneity in pad test among eligible studies was not significant (I^2^ < 0.001%; *P* = .76).

### Outcomes

#### International Consultation on Incontinence Questionnaire–Short Form:

**Figure 4.**
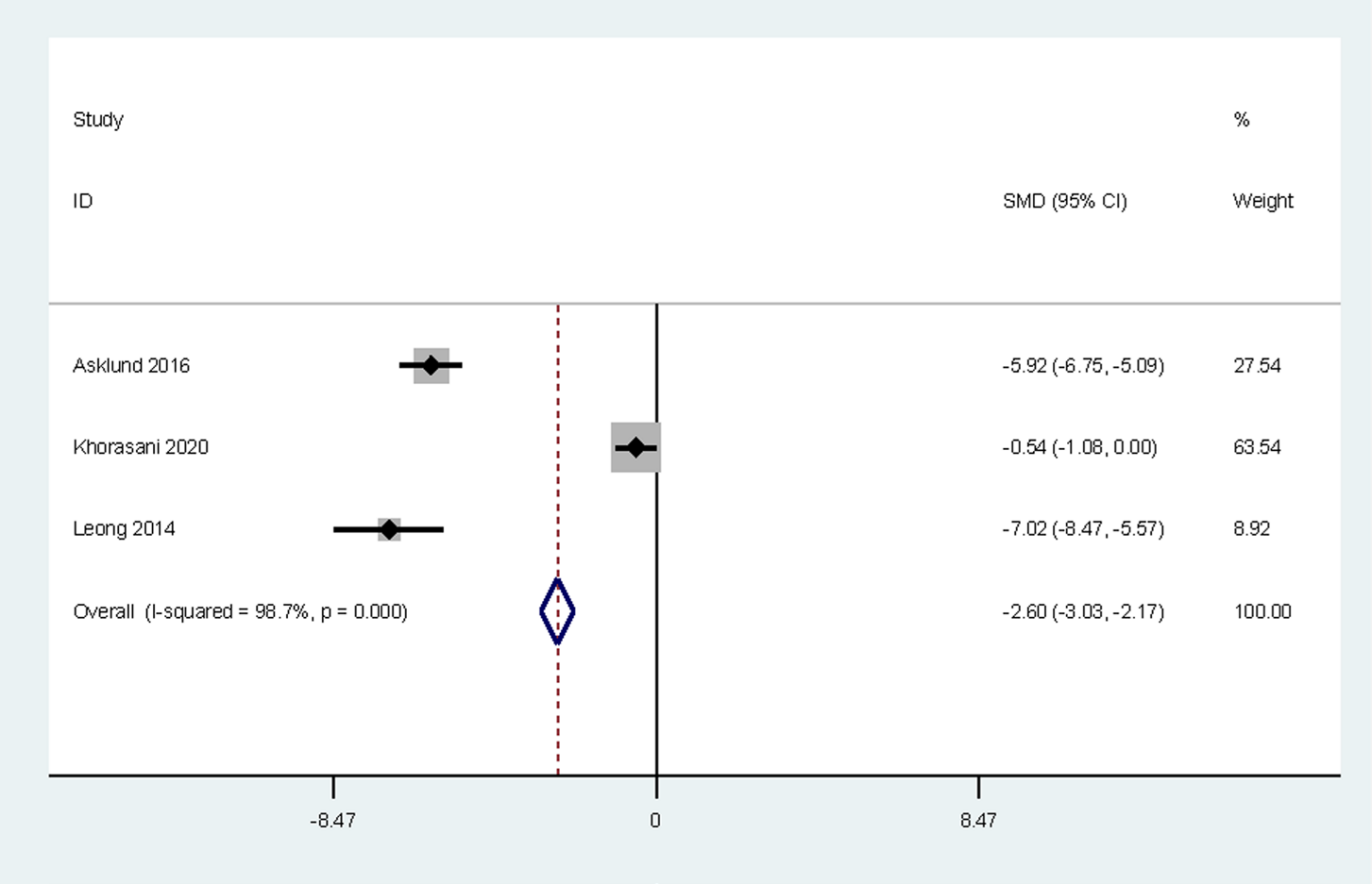
Difference in urinary incontinence severity according to the ICIQ-SF between physiotherapy and comparison groups. ICIQ-SF, Incontinence Impact Questionnaire–Short Form.

Twelve studies used the ICIQ-SF to assess UI severity.^17,28,30,36,38,40,43,45,50,52,53,57^ Five of them reported significantly different results between treatment and control groups,^40,43,45,50,53^ but the rest of them reported no differences between the 2 groups.^17,28,30,36,38,52,57^ Three studies entered the meta-analysis.^17,42,45^ The results of assessing urinary incontinence severity by ICIQ-SF showed statistically significant differences between physiotherapy and comparison groups [SMD: −2.60; 95% CI (−3.03, −2.17); *P* < .0001]. In addition, heterogeneity in studies was very high [I^2^:98.7%; *P* < .0001] (Figure 4).

#### Pad Test:

**Figure 5.**
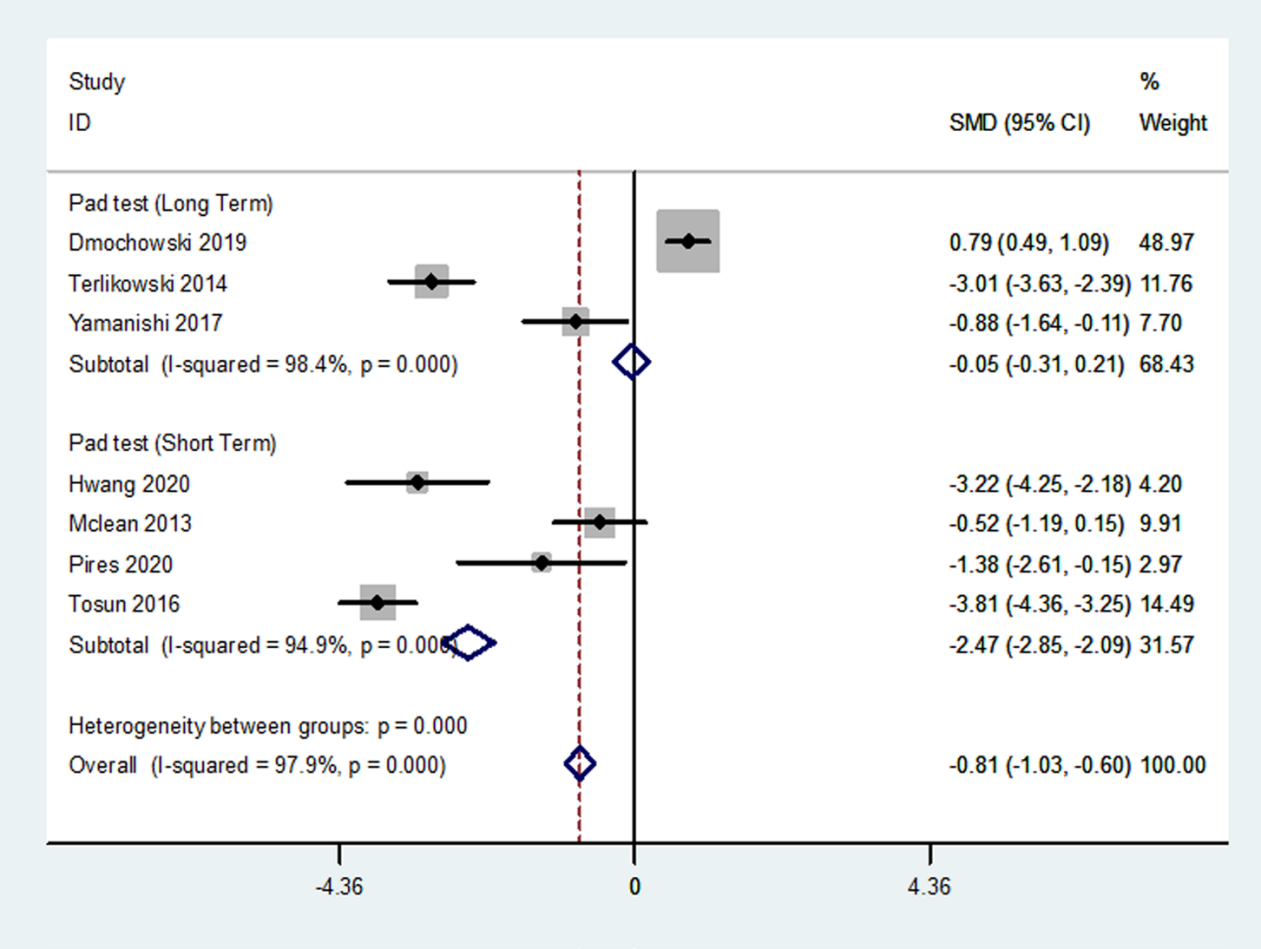
Difference in urinary incontinence severity according to pad test between physiotherapy and comparison groups.

Eleven studies reported pad test results.^29-31,33,37,41,48,53-56^ Among them, 4 showed no differences between treatment and control groups.^30,41,48,54^ The remaining 7 studies showed better results in the treatment group compared with the control one.^29,31,33,37,53,55,56^ Seven studies were included in the meta-analysis.^33,37,41,53-56^ The results of assessing urinary incontinence severity according to long-term pad test showed statistically significant differences between physiotherapy and comparison groups [SMD: −0.05; 95% CI (−0.31, 0.21); *P *< .0001]. In addition, heterogeneity in studies was very high [I^2^:98.4%, *P* < .0001] (Figure 5).

In addition, according to short-term pad test, there was a statistically significant difference between physiotherapy and comparison groups [SMD: −2.47; 95% CI (−2.85, 2.09); *P* < .0001]. However, heterogeneity was very high [I^2^: 97.9%; *P* < .0001] (Figure 5).

#### Pelvic Floor Muscle Function

Six studies used the Oxford Scale to assess PFM strength.^17,32,40,44,48,56^ Only 2 of them reported a difference between treatment and control groups.^17,56^

**Figure 6.**
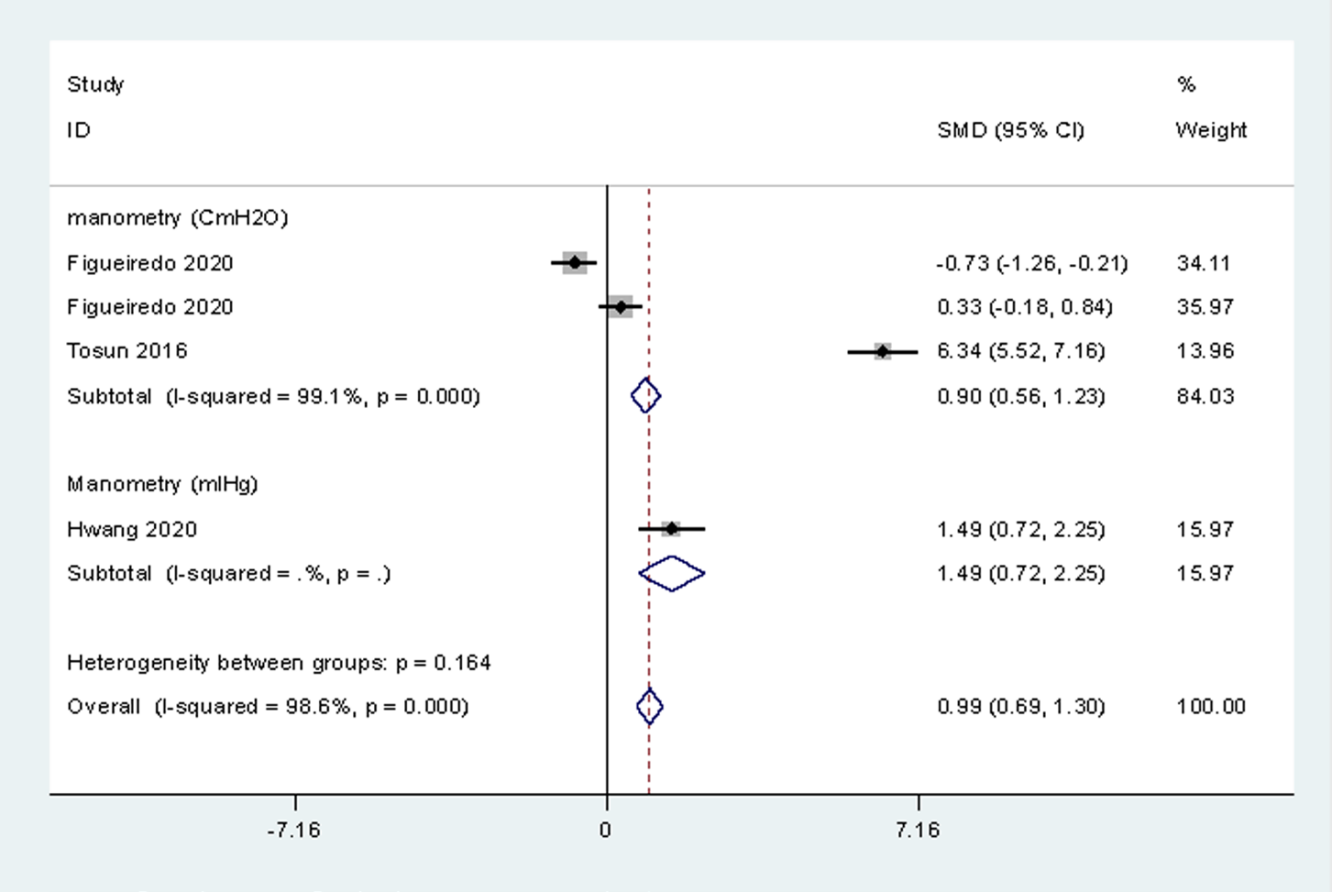
Difference in PFM function according to manometry between physiotherapy and comparison groups. PFM, pelvic floor muscle.

Besides, 7 studies assessed PFM strength due to manometry.^30,33,37,46-48,55^ Four of them reported significant differences between treatment and control groups.^33,46,47,55^ Three of them used manometry, which were included in the meta-analysis.^37,46,55^ According to manometry, there was a statistically significant difference between physiotherapy and comparison groups [SMD: 0.99; 95% CI (0.69, 1.30); *P* < .0001]. In addition, heterogeneity was very high [I^2^: 98.6%; *P* < .0001] (Figure 6).

#### Improvement

**Figure 7.**
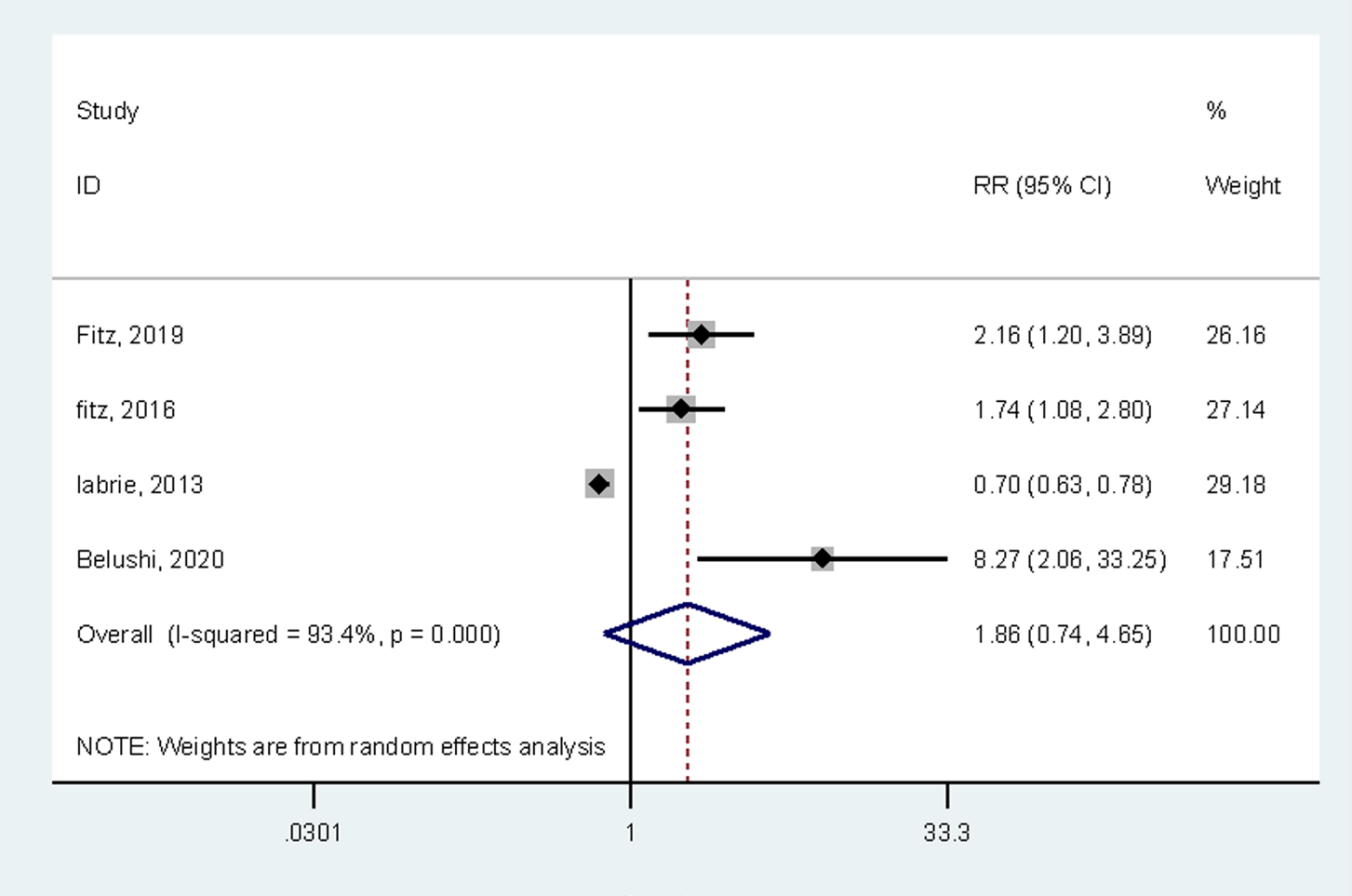
Difference in improvement between physiotherapy and comparison groups.

Thirteen studies reported cure/improvement.^31,32,35,42-45,47-49,52,54,57^ Of these, 8 reported a significant different improvement between treatment and control groups.^31,32,35,42,43,45,48,57^ Three studies were included in the meta-analysis regarding improvement,^31,43,48^ which showed statistically significant difference between physiotherapy and comparison groups [RR: 1.86; 95% CI (0.74, 4.65); *P* < .0001]. Nonetheless, heterogeneity in studies was very high [I^2^: 93.4%; *P* < .0001] (Figure 7).

#### Grade

Based on the grade, studies entered the meta-analysis with 5 main outcomes, and there was serious inconsistency between them (Table 3). Therefore, the grade of recommendation of included studies in meta-analysis was very low in all 5 outcomes. There are a large number of RCTs regarding the effect of physiotherapy and pelvic floor exercises on SUI. Despite the fact, there is much heterogeneity in the intervention and control groups owing to different tools and methods of measuring the consequences.

## Discussion

The main objective of this systematic review and meta-analysis was to analyze RCTs that investigated the effects of physiotherapy techniques such as BF, PFMT, and ES or surgical treatments and so on on episodes of urinary loss, QOL, and muscle strength in SUI patients. The main finding was that physiotherapy treatment successfully improves both subjective and objective measures in women with SUI compared with control groups or other treatments. However, owing to heterogeneity of studies, there is lack of consensus about appropriate treatment parameters for women with SUI.

Our analysis included 3 studies related to incontinence severity^17,42,45^ presenting favorable results for physiotherapy treatment over comparison groups. The effect size estimated for 2 studies^42,45^ supports the positive effects of physiotherapy but effect size of 1 study^17^ was small and not significant. Huge variation in PFMT programs, varying from supervised PFMEs^42^ to mobile app^45^ and home-based stabilization exercises with focus on PFMs,^17^ and study populations including women with postnatal SUI^17^ and elderly population^42^ were found among the selected studies. Despite these differences, supervised and unsupervised PFMT were always superior to the control intervention.

Previous studies reported that combined BF and PFMT have multiple effects, including increased trophism and neuromuscular function of the pelvic floor muscles,^58^ which is effective in improving urethral closure during increase of intra-abdominal pressure.^59^ However, improvement analysis after physiotherapy treatment including PFMT^31,35,43^ and BF^48^ compared with control groups showed nonsignificant results. Adding BF to the PFMT did not have any additional advantages in some investigations^47,48^ but was better than using BF alone in another study.^55^

The results mentioned above for subjective and objective outcomes of incontinence severity, pad test, PFM function, and improvement may indicate favorable response regarding the physiotherapy treatments despite high level of heterogeneity. Different parameters of PFMT, duration of treatment, and variety of adjunct treatment to PFMT may lead to significant heterogeneity. Overall, physiotherapy for SUI focuses on PFMT alone or combined with ES and BF.

Khorasani et al^17^ reported the importance of supervised program to increase adherence of patients to treatment and monitor their performance. Figueiredo et al^46^ compared supervised PFMT with group training and individualized training progressing to group training and reported superior PFM function in the latter. Tosun et al^37^ reported an improvement in PFM function after a 12-week individualized home program which was progressed based on the PERFECT scheme. Asklund et al^45^ presented an app-based PFMT program through which the patient could progress through his or her training program. This study indicated that non–face-to-face PFMT may be an effective treatment for SUI.^45^ Ghaderi et al^31^ reported that PFMT focusing on strength, endurance, and progression of training in different positions may be a safe and effective treatment for women with all kinds of UI. 

Altogether, it can be interpreted that a progressive and intensive PFMT program focusing on endurance and strength of PFMs in different positions is key in the conservative treatment of SUI. It seems that in most effect studies of physiotherapy for urinary incontinence, PFMT has been studied. Besides, the effect of adding other treatment modalities to PFMT has been investigated widely.^28,31,32,49,52^ The superiority of PFMT was indicated comparing intensive and supervised PFMT, with any other modality or method as control group.^17,28,32,34,37,41,45^ Adding stabilization exercises to PFMT increased the effectiveness of training in the long run,^49,50^ but adding adductor muscles exercises to PFMT did not show any additional effects.^52^

Moreover, individualized PFMT with a supervising physiotherapist was preferred when comparing PFMT as home training, only based on previous training and education or using a mobile application with individualized and supervised PFMT.^31,42^ However, in terms of cost-effectiveness, Internet-based training or the use of mobile apps may be better than no treatment.^28,34,38,60^ Using an application to do exercises^38,45^ or remote-control exercises^36^ showed better results than home-based exercises without any supervision or no treatment.^38^ Treatment sessions alone without supervision of exercises did not replace supervised sessions or change adherence of patients to treatment.^40^

Overall, PFMT is an effective first-line treatment modality for SUI. If possible and affordable, performing individual PFMT under the supervision of a well-trained physiotherapist at least once a week is the treatment of choice. Biofeedback therapy as an adjunct to PFMT may be useful in patients with no or insufficient awareness of their PFMs.^28,32,47^ Based on this systematic review and other available literature, so far, the use of surface or intravaginal ES cannot be recommended for patients with SUI.^54-56^

However, if supervised physiotherapy is not available, it is recommended to perform home PFMT using educational pamphlets or online applications.^36,38,44,45^

### Limitations and strengths

There are some limitations to our systematic review that should be acknowledged. This study included articles only in Persian or English. Also, included studies were highly heterogeneous in terms of study population, treatment protocol, and duration of treatments. Accordingly, there is a need for a well-designed study with more consistent treatment protocols and study populations. In fact, the results of this study should be implicated with caution. However, the strength of our study is that almost all types of physiotherapy treatments, including PFMT, BF, and ES, were evaluated. Based on this review, physiotherapy for SUI could be updated after 8 years.^20^

## Conclusion

Although physiotherapy treatments showed significant results over comparison groups, there was considerable statistical heterogeneity among the eligible studies. Further RCTs should assess long-term effects of physiotherapy treatments in women with SUI. Moreover, further studies should investigate which treatment parameters are more practical and effective. Finally, it is recommended to use PFMT as a first-line treatment in women with SUI to improve both subjective and objective outcomes. Biofeedback therapy also can be used as an adjunct to PFMT to improve treatment results. Results of using ES in women with SUI are not conclusive and more studies are required.

## Data Availability:

The datasets generated during and/or analyzed during the current study are available from the corresponding author on reasonable request.

## Figures and Tables

**Table 1.** Critical Appraisal of Eligible RCTs

Study	Q1	Q2	Q3	Q4	Q5	Q6	Q7	Q8	Q9	Q10	Q11	Q12	Q13	Quality	Overall Appraisal
Ahlund 2013 ^44^	Y	Y	Y	U	U	Y	N	Y	N	Y	Y	N	Y	**	Include
Belushi 2020 ^43^	Y	Y	Y	N	N	Y	Y	Y	Y	Y	Y	Y	Y	*	Include
Asklund 2016 ^45^	Y	Y	Y	N	N	U	Y	Y	Y	Y	Y	Y	Y	**	Include
Bertotto 2017 ^28^	Y	Y	Y	N	N	N	Y	N	Y	Y	Y	Y	Y	**	Include
Carrión Pérez 2015 ^61^	N	U	Y	U	U	Y	N	N	Y	N	N	Y	Y	***	Exclude
Oliveira 2019 ^30^	Y	Y	Y	N	N	Y	Y	Y	U	Y	U	U	Y	**	Include
Abreu 2017 ^49^	Y	U	Y	U	U	Y	Y	N	N	Y	Y	Y	Y	**	Include
Dmochowski 2019 ^54^	Y	Y	Y	N	N	Y	N	Y	Y	Y	Y	Y	N	**	Include
Ulla Due 2018 ^52^	Y	Y	Y	N	Y	N	Y	Y	U	Y	Y	Y	Y	**	Include
Sandra Engberg 2016 ^62^	N	N	N	N	N	N	Y	Y	N	N	U	N	N	***	Exclude
Figueiredo 2020 ^46^	Y	Y	Y	N	N	Y	Y	N	N	Y	Y	Y	Y	**	Include
Fitz 2019 ^31^	Y	Y	Y	N	N	N	Y	Y	Y	Y	Y	Y	Y	**	Include
Fitz 2017 ^48^	Y	Y	Y	N	N	Y	Y	Y	Y	Y	Y	Y	Y	*	Include
Ghaderi 2016 ^19^	U	U	Y	U	U	U	Y	N	N	Y	Y	Y	Y	***	Exclude
Hwang 2020 ^55^	Y	N	Y	N	Y	N	Y	Y	N	Y	U	Y	Y	**	Include
Kaya 2014 ^32^	Y	Y	Y	Y	Y	Y	Y	Y	N	Y	Y	Y	Y	*	Include
Khorasani 2020 ^17^	Y	Y	Y	U	U	Y	Y	Y	Y	Y	Y	Y	Y	*	Include
Labrie 2013 ^35^	Y	N	Y	U	U	U	Y	N	Y	Y	Y	Y	Y	**	Include
Leong 2014 ^42^	Y	Y	Y	N	N	N	Y	Y	N	Y	Y	Y	Y	**	Include
Manonai 2015 ^47^	Y	U	Y	N	N	Y	Y	Y	Y	Y	Y	Y	Y	**	Include
McLean 2013 ^41^	Y	U	Y	Y	U	U	Y	Y	N	Y	Y	Y	Y	**	Include
Okayama 2019 ^57^	U	U	Y	N	N	Y	Y	Y	Y	Y	Y	Y	Y	**	Include
Pereira 2012 ^29^	Y	Y	Y	N	N	U	N	Y	N	Y	Y	Y	Y	**	Include
Pires 2020 ^33^	Y	Y	Y	Y	U	N	Y	Y	Y	Y	Y	Y	Y	*	Include
Ptak 2017 ^50^	Y	Y	Y	N	N	N	Y	Y	N	Y	Y	Y	Y	**	Include
Sacomori 2015 ^40^	Y	N	Y	N	N	U	Y	Y	Y	Y	Y	Y	Y	**	Include
Sjöström 2017^38^	U	U	Y	U	U	U	Y	Y	N	Y	Y	Y	Y	**	Include
Sjostrom 2015^39^	U	U	Y	N	N	N	N	Y	N	Y	Y	Y	N	***	Exclude
Sjöström 2013^63^	Y	U	Y	N	N	N	N	Y	Y	Y	Y	Y	N	***	Exclude
Terlikowski 2013 ^56^	Y	Y	Y	Y	Y	Y	Y	Y	N	Y	Y	Y	Y	*	Include
Tosun 2016 ^37^	Y	Y	Y	U	N	U	Y	Y	N	Y	Y	Y	Y	**	Include
Vural 2013 ^64^	N	N	N	N	N	N	N	N	N	Y	Y	Y	N	***	Exclude
Wang 2016 ^65^	U	U	Y	N	N	N	N	N	N	Y	Y	Y	N	***	Exclude
Wang 2019 ^36^	Y	Y	Y	U	U	Y	Y	N	Y	Y	Y	Y	Y	**	Include
Weber-Rajek 2019 ^34^	Y	Y	Y	U	Y	U	Y	Y	N	Y	Y	Y	Y	**	Include
Yamanishi 2017 ^53^	Y	N	Y	Y	U	U	Y	N	N	Y	Y	Y	Y	**	Include
**Total %**	**56.25**	**50**	**87.5**	**56.25**	**87.5**	**93.75**	**93.75**	**81.25**	**93.75**						

JBI critical appraisal checklist for randomized controlled trials.

JBI, Joanna Briggs Institute; N, no; RCTs, randomized controlled trials; U, unclear; Y, yes.

**Table 2.** Characteristics of Included Clinical Trials

Author (Ref)/Country/Year/Design	Total Sample Size/Study Subgroup	Group Characteristics Sample’s Number (Mean Age)/Intervention Description					Study Duration	Methods for Outcome Measurement
		Control	Intervention					
Ahlund et al^44^ Sweden 2013 RCT	n = 100 SUI women 10-16 weeks after their first baby	n = 49 (33) Written postpartum instructions	n = 49 (33) PFM exercise program				12 weeks	MVC (cmHg)
								Endurance (seconds)	Oxford Scale	ICIQ-FLUTS (Incontinency score)
								Belushi et al^43^ Oman 2020 RCT	n = 73 SUI women	n = 37 (34.30) group lecture on anatomy and physiology of pelvic floor for 15 minutes	n = 36 (35.69) Lecture on anatomy and physiology of pelvic floor/ strength and endurance training with weekly reminder				12 weeks	ICIQ-SF Arabic version
																Power graded by MOGS and endurance based on time of sustained contraction
Asklund et al^45^ Sweden 2016 RCT	n = 123 SUI women	n = 61 (44.7) Postponed treatment	n = 62 (44.8) Mobile app including PFMT (3 times a day, endurance and strengthening)				12 weeks	ICIQ-UI SF
								PGI-I
Bertotto et al^28^ Brazil 2017 RCT (3 groups)	n = 49 (57.1) postmenopausal women with SUI	n = 14 (59.3) No treatment	1			2	8 weeks	Modified Oxford grading
			n = 15 PFME			n = 16 PFME + BF		ICIQ-SF
Oliveira et al^30^ Brazil 2019 RCT	n = 22 Women with MUI	n = 11 (49.36) PFMT	n = 11 (51.50) PFMT + Weight loss PFMT (breathing, pelvic mobility, and abdominal exercise) Weight loss (three meetings with nutritionist)				8 weeks	Urinary loss (ICIQ-SF)
								Manometry (cmH_2_O)	1 hour Pad test
								Abreu et al^49^ Brazil 2017 RCT	n = 33 Women with SUI	n = 16 (50.6) PFM exercise	n = 17 (57.3) Dynamic lumbopelvic stabilization				5 weeks	Frequency of UI
PGI-I															
Dmochowski et al^54^ USA 2019 Randomized control noninferiority trial	n = 180 SUI women	n = 89 (45.9) Neuromuscular external stimulation device (NMES)	n = 91 (47.8) FDA-approved intravaginal device				12 weeks	Pad weight test
								Incontinence QOL (I-QOL)	PGI-I
								Due et al^52^ Denmark 2018 RCT	n = 41 SUI women	n = 17 (45) PFMT	n = 14 (44) Occlusion training of a thigh (KAATSU) + PFMT				12 weeks	ICIQ-UI SF
PGI-I															
Figueiredo et al^46^ Brazil 2020 RCT (3 groups)	n = 90 SUI women	n = 30 (50.3) Individual training (IT)	1		2		12 weeks	PFM function (POWER scale)
			n = 30 (50.8) Individual progressing to group training (IPGT)		n = 30 (57.8) Group training			
								PFM manometry (cmH_2_O)
Fitz et al^31^ Brazil 2019 RCT (parallel designed)	n = 69 Predominant SUI women	n = 35 (56) Home PFMT	n = 34 (57.7) Outpatient + home PFMT Outpatient (PFMT in supine/sitting and standing position) PFMT (in accordance with Consensus on Exercise Reporting Template)				12 weeks	Objective cure of SUI (modified 20-minute pad test)
Fitz et al^48^ Brazil 2016 RCT	n = 72 Women with SUI	n = 37 (56.6) Outpatient PFMT + home PFMT	n = 35 (56.1) Outpatient BF + home PFMT				12 weeks	PFM function (Oxford Grading Scale)
								Manometry (cmH_2_O)	Objective cure (pad test)
								Hwang et al^55^ South Korea 2020 RCT	n = 34 Patient with SUI	n = 17 (41.1) Daily walking over 20 minutes	n = 17 (42.3) surface electrical stimulation (SES)				8 weeks	Manometry (mmHg)
Pad test	UDI-6														
Kaya et al^32^ Turkey 2014 RCT	n = 108 Women with SUI, urgency (UUI), or mixed (MUI)	n = 52 (50.9) BT (bladder training)	n = 56 (48.7) BT (bladder training) + PFMT				6 weeks									Oxford Scale
								UDI-6	Global rating of improvement
								Khorasani et al^17^ Iran 2020 RCT (parallel)	n = 80 Postnatal SUI and LBP	n = 27 (30.25) No treatment	n = 27 (30.75) Home-based stabilization exercises focusing on pelvic floor muscles				12 weeks	Oxford Scale
ICIQ-UI SF															
Labrie et al^35^ The Netherlands 2013 RCT	n = 460 Women with SUI	n = 230 (50.2) Surgery group retropubic and trans obturator midurethral-sling surgical techniques	n = 230 (50) Physiotherapy group: PFMT					PGI-I
Leong et al^42^ Hong Kong China 2014 Controlled trial	n = 55 Women with mild-to-moderate urinary incontinence Aged over 65 years	n = 27 (75.4) Advice and pamphlet about management of UI	n = 27 (73) Urinary Continence Physiotherapy Program (UCPP) involving education and exercise				12 weeks	The number of UI episodes in the previous 7 days (UI7)
								Incontinence Impact Questionnaire–Short Form (IIQ-7)	Subject perception of improvement
								Manonai et al^47^ Thailand 2015 RCT	n = 61 Women with SUI	n = 32 (48.50) PFMT	n = 29 (46.96) PFMT + BF				16 weeks	a. Vaginal squeeze pressure patient-based
Three-point symptom severity scale															
Mclean et al^41^ Canada 2013 RCT	n = 40 Women with SUI	n = 17 (54) No treatment	n = 18 (49.5) Weekly physiotherapy session and progressive home PFM exercises				week	a. Leakage episode
								b. Pad test
Okayama et al^57^ Japan 2019 RCT	n = 89 Women with SUI	n = 28 (43.5) No treatment	1	2			12 weeks	UI episodes
			n = 30 (44.0) Shaper	n = 31 (45.0) PFMT				ICIQ-SF
Pereira et al^29^ Brazil 2012 RCT	n = 45 Postmenopausal women with SUI	n = 15 (62.0) No treatment	1	2			13 weeks	Pad test
			n = 15 (64.0) Vaginal weights	n = 15 (62.0) PFMT				Strength (device)
								Satisfaction
Pires et al^33^ Portugal 2020 RCT	n = 14 Volleyball athletes with SUI	n = 6 (21.83) No treatment	n = 7 (22.71) PFMT				16 weeks	Pad test
								Vaginal resting pressure
Ptak et al^50^ Poland 2017 RCT	n = 140 Women with SUI	n = 70 (53.0) PFMT	n = 70 (53.1) PFMT+ transversus abdominis contraction				3 months	ICIQ-LUTSqol
Sacomori et al^40^ Brazil 2015 randomized trial	n = 86 (50) Women with UI	n = 43 Three physiotherapy sessions (educated about UI, awareness exercise, correct contraction, UI exercise folder)	n = 43 Three physiotherapy sessions (educated about UI, awareness exercise, correct contraction, UI exercise folder) received self-efficacy information in addition to the mentioned physiotherapy sessions (reminder and modeling video)				90 days	ICIQ-SF
								Oxford Scale
Sjostorm et al^38^ Sweden 2017 RCT	n = 123 Community-dwelling women with SUI	n = 61 (44.7) No treatment	n = 62 (44.8) Tät app (PFMT program with 6 basic and 6 advanced level)				3 months	ICIQ-UI SF
								ICIQ-LUTSqol
Terlikowski et al^56^ Poland 2013 RCT	n = 102 Women with SUI	n = 29 (45.6) Placebo ES and BF, and skills and strategies for preventing incontinence	n = 64 (46.9) TVES + BF and skills and strategies for preventing incontinence				16 weeks	Oxford Scale
								24 hour pad test
Tosun et al^37^ Turkey 2016 RCT	n = 116 Incontinent women	n = 70 (49.6) No treatment	n = 70 (51.7) PFMT				12 weeks	Pad test (g)
								Perineometer (cmH_2_O)
Wang et al^36^ China 2019 RCT	n = 108 Primiparas with SUI	n = 54 (29.1) A 45-minute pelvic floor rehabilitation education	n = 54 (29.2) A 45-minute pelvic floor rehabilitation education + audio guidance training (PFMT)				3 months	ICIQ-SF
Weber-Rajek et al^34^ Poland 2019 RCT	n = 49 Overweight or obese elderly women with SUI	n = 21 (35) No treatment	n = 28 (31.75) PFMT				4 weeks	UI severity
Yamanishi et al^53^ Japan 2017 (online publication) RCT	n = 39 Women with UI	n = 12 Sham intervention	n = 18 Magnetic stimulator				10 weeks	IEF
								ICIQ-SF	Pad test

BF, biofeedback; ES, electrical stimulation; ICIQ-LUTSqol, International Consultation on Incontinence Questionnaire–Lower Urinary Tract Symptoms, Quality of Life; ICIQ-SF, International Consultation on Incontinence Questionnaire–Short Form; ICIQ-UI SF, International Consultation on Incontinence Questionnaire–Urinary Incontinence, Short Form; IEF, incontinence episode frequency; LBP, low back pain; MVC, maximally voluntary contraction; PFM, pelvic floor muscle; PFME, pelvic floor muscle exercises; PFMT, pelvic floor muscle training; PGI-I, Patient Global Impression of Improvement; QOL, quality of life; SUI, stress urinary incontinence; TVES, transvaginal electrical stimulation; UI, urinary incontinence.

**Table 3.** Levels and Grades of Included Studies

Quality Assessment							SMD (95% CI)	Certainty
No. of Studies	Design	Risk of Bias	Inconsistency	Indirectness	Imprecision	Publication Bias		
**Urinary Incontinence Severity According to ICIQ-SF**								
3	Randomized trials	No serious risk of bias	Very serious inconsistency^1^	No serious indirectness	*Serious imprecision* ^2^	Undetected	−2.60 (−3.03, −2.17)	Very low ⨁OOO
**Urinary Incontinence Severity According to Pad Test**								
7	Randomized trials	No serious risk of bias	Very* serious* *inconsistency* ^1^	No serious indirectness	*Serious* *imprecision^2^*	Undetected	−0.05 (−0.31, 0.21)	Very low ⨁OOO
**PFM Function According to Oxford Scale**								
3	Randomized trials	No serious risk of bias	Very* serious* *inconsistency* ^1^	No serious indirectness	*Serious* *imprecision^2^*	Undetected	0.94 (0.62, 1.26)	Very low ⨁OOO
**PFM Function According to Manometry**								
4	Randomized trials	No serious risk of bias	Very* serious* *inconsistency* ^1^	No serious indirectness	*Serious* *imprecision^2^*	Undetected	0.99 (0.69, 1.30)	Very low ⨁OOO
**Improvement**								
4	Randomized trials	No serious risk of bias	Very* serious* *inconsistency* ^1^	No serious indirectness	*Very serious* *imprecision^2^*	Undetected	RR: 1.86 (0.74, 4.65)	Very low ⨁OOO

ICIQ-SF, International Consultation on Incontinence Questionnaire–Short Form; PFM, pelvic floor muscle; SMD, standarized mean difference.

^1^Considerable inconsistency; ^2^total event is less than 300.
